# Octopus Cells in the Posteroventral Cochlear Nucleus Provide the Main Excitatory Input to the Superior Paraolivary Nucleus

**DOI:** 10.3389/fncir.2017.00037

**Published:** 2017-05-31

**Authors:** Richard A. Felix II, Boris Gourévitch, Marcelo Gómez-Álvarez, Sara C. M. Leijon, Enrique Saldaña, Anna K. Magnusson

**Affiliations:** ^1^Unit of Audiology, Department of Clinical Science, Intervention and Technology, Karolinska InstitutetStockholm, Sweden; ^2^Institut Pasteur, Unité de Génétique et Physiologie de l'AuditionParis, France; ^3^Institut National de la Santé et de la Recherche Médicale, UMRS 1120Paris, France; ^4^Université Pierre et Marie CurieParis, France; ^5^Neuroscience Institute of Castilla y León (INCyL), Universidad de SalamancaSalamanca, Spain; ^6^Institute of Biomedical Research of Salamanca (IBSAL)Salamanca, Spain

**Keywords:** auditory brainstem, temporal processing, tract tracing, calretinin, cluster analysis

## Abstract

Auditory streaming enables perception and interpretation of complex acoustic environments that contain competing sound sources. At early stages of central processing, sounds are segregated into separate streams representing attributes that later merge into acoustic objects. Streaming of temporal cues is critical for perceiving vocal communication, such as human speech, but our understanding of circuits that underlie this process is lacking, particularly at subcortical levels. The superior paraolivary nucleus (SPON), a prominent group of inhibitory neurons in the mammalian brainstem, has been implicated in processing temporal information needed for the segmentation of ongoing complex sounds into discrete events. The SPON requires temporally precise and robust excitatory input(s) to convey information about the steep rise in sound amplitude that marks the onset of voiced sound elements. Unfortunately, the sources of excitation to the SPON and the impact of these inputs on the behavior of SPON neurons have yet to be resolved. Using anatomical tract tracing and immunohistochemistry, we identified octopus cells in the contralateral cochlear nucleus (CN) as the primary source of excitatory input to the SPON. Cluster analysis of miniature excitatory events also indicated that the majority of SPON neurons receive one type of excitatory input. Precise octopus cell-driven onset spiking coupled with transient offset spiking make SPON responses well-suited to signal transitions in sound energy contained in vocalizations. Targets of octopus cell projections, including the SPON, are strongly implicated in the processing of temporal sound features, which suggests a common pathway that conveys information critical for perception of complex natural sounds.

## Introduction

Sensory processing relies on merging information from various stimulus features into streams, and deciphering complex sounds, such as human speech, requires delicate analysis of both spectral and temporal cues. The information in each stream is then conveyed to higher order areas, where combinations (Portfors and Wenstrup, 2002; Suga, 2015) of increasing complexity are formed (Leaver and Rauschecker, 2010; Overrath et al., 2015). The auditory cortex plays a crucial role in the perception of auditory objects (Griffiths and Warren, 2004; Hickok and Poeppel, 2007), but little is known of the contributions of subcortical pathways. At the level of the brainstem, different patterns of spiking activity have been linked to both spectral (Moore and Cashin, 1976; Pressnitzer et al., 2008) and temporal (Rupert et al., 1977; Sayles and Winter, 2008) acoustic features of complex sounds. For instance, octopus cells in the cochlear nucleus (CN) are especially well-suited for extracting temporal information due to their broad frequency tuning (Golding et al., 1995), unsurpassed capabilities of following broadband transients (Oertel et al., 2000), and synchronous responses to amplitude-modulated (Rhode and Greenberg, 1994) and formant-like sounds (Rhode, 1998). These findings have led to the suggestion that the octopus cells extract and convey information relevant for speech segmentation (Oertel, 2005), but our understanding of how this information is processed further in the brainstem is lacking.

One target of octopus cells is the superior paraolivary nucleus (SPON) located prominently in the superior olivary complex (Zook and Casseday, 1985; Friauf and Ostwald, 1988; Thompson and Thompson, 1991; Schofield, 1995; Saldaña et al., 2009). The SPON has been implicated as an early brainstem region specialized for extracting sound features contained in vocal communication. This notion is supported by the fact that, like octopus cells, SPON neurons are well-suited for the extraction of coarse temporal cues important for speech processing. Similar to octopus cells, many SPON neurons have well-timed transient spiking to the onset of sounds, in addition to their well-documented and prominent spiking to the sound offset (bat: Grothe, 1994; rabbit: Kuwada and Batra, 1999; gerbil: Behrend et al., 2002; Dehmel et al., 2002; rat: Kulesza et al., 2003; mouse: Felix et al., 2013, 2015). This on-off spiking behavior is thought to convey coarse temporal sound structure, such as abrupt changes in sound energy (Kulesza et al., 2003; Kadner and Berrebi, 2008), silent gaps in ongoing sounds (Kopp-Scheinpflug et al., 2011), and the temporal envelope of sounds (Grothe, 1994; Kuwada and Batra, 1999; Felix et al., 2011, 2013).

Progress has been made in investigating the mechanisms and functional implications of the SPON offset response, which is generated primarily by a post-inhibitory rebound mechanism (Felix et al., 2011; Kopp-Scheinpflug et al., 2011), but the precise origin(s) and role of the putative excitatory input to the SPON responsible for onset spiking is presently unknown. Most studies of auditory brainstem anatomy suggest that excitatory inputs to the SPON arise from a mixed neuronal population in the posteroventral CN (PVCN; Zook and Casseday, 1985; Friauf and Ostwald, 1988; Thompson and Thompson, 1991; Schofield, 1995; Saldaña et al., 2009) consisting of octopus and multipolar cells, while an additional input from bushy cells of the anteroventral cochlear nucleus (AVCN) has also been proposed (Saldaña et al., 2009). The physiological properties of SPON neurons have not clarified either the types or relative contributions of excitatory inputs. For instance, excitatory synaptic inputs to SPON neurons undergo developmental pruning resulting in a few strong fibers with high release probability (Felix and Magnusson, 2016) compatible with a dominant octopus cell input (Godfrey et al., 1975; Ritz and Brownell, 1982; Rhode and Smith, 1986). However, SPON neurons also exhibit multiple spiking patterns in response to intrinsic depolarization *in vitro* (Felix et al., 2013), and it is unclear whether variations in onset spiking to sound stimulation *in vivo* reflect one or multiple excitatory inputs.

Inhibition that originates from SPON projections enhances the extraction of coarse temporal features of complex sounds in the inferior colliculus (IC) at the level of the midbrain (Felix et al., 2015). Synaptic inhibition in the IC, which is an important site of temporal processing en route to the cortex, has also been shown to increase neuronal selectivity to vocalizations (Mayko et al., 2012). Given the importance of this pathway, investigation of the precise nature of excitatory inputs underlying the onset response of SPON neurons is needed. In this study we provide detailed information of excitatory input to the SPON of the mouse and its origin by combining retrograde tract tracing and immunolabeling with statistical clustering of stochastic excitatory events of SPON neurons. We compared the characteristics of SPON inputs with those of principal and non-principal neurons of the adjacent lateral superior olive (LSO), both of which potentially receive two sources of excitatory inputs (Sterenborg et al., 2010; Gómez-Álvarez and Saldaña, 2016). Taken together, this multi-disciplinary approach leads us to conclude that octopus cells provide the main excitatory projection that drives the onset spiking of SPON neurons.

## Materials and methods

This study was carried out in accordance with the recommendations of the EC Council Directive (2010/63/EU) and was approved by the local Animal Care and Use Committees in Sweden (Permit N52/13) and Spain (Permit associated to grant PI10/01803).

We utilized animals with a broad range of ages. We believe that the different ages would not substantially alter the anatomical and physiological properties of the hardwired circuits examined (Leijon et al., 2016). This notion is based on our previous studies demonstrating that there is no qualitative change of the excitatory inputs to the SPON over the age range covering the postnatal development of hearing (Felix and Magnusson, 2016), and the fact that we have observed a robust onset response to broad band sounds in adult mice *in vivo* (Felix et al., 2013).

### Anatomical tract tracing

For the surgical injection of biotinylated dextran amine (BDA) and Fluoro-Gold (FG) tracers, young adult female mice (BDA: *n* = 4, ~P60, 25 g; FG: *n* = 3, ~P30, 22 g) were deeply anesthetized with a mixture of ketamine (80 mg/kg body weight) and xylazine (6 mg/kg body weight) administered intraperitoneally. For the transcardial perfusion of fixatives, the animals were deeply anesthetized with an overdose of sodium-pentobarbital. We used the bidirectional neuroanatomical tracer BDA [10,000 MW; Molecular Probes (Invitrogen) product D-1956; Eugene, OR] injected as a 10% solution in 0.1 M sodium phosphate buffer and the retrograde tracer FG (Fluorochrome; Denver, CO) injected as a 2% solution in 0.2 M sodium acetate buffer. Under stereotaxic guidance, glass micropipettes (5–10 μm inner diameter at the tip) loaded with the tracers were inserted into the SPON of deeply anesthetized mice. To avoid damage to the prominent transverse sinus, the pipettes were lowered into the brain via a dorsocaudal to ventrorostral approach, forming a 15° angle with the coronal plane. The tracer was delivered by iontophoresis using a pulsed 3.5 μA DC positive current (7 s on/7 s off) for 10–15 min (BDA) or 3.0 μA for 1–5 min (FG).

Following 5 days of survival, the mice were again anesthetized deeply and their brains fixed by transcardial perfusion of buffered 4% formaldehyde. After cryoprotection in 30% sucrose in phosphate buffer, the brains were cut coronally on a freezing microtome at a thickness of 40 μm. To visualize the BDA tracer, the sections were processed by the avidin-biotin-peroxidase complex procedure (ABC, Vectastain, Vector Labs, Burlingame, CA), followed by standard histochemistry for peroxidase with heavy-metal intensification (e.g., Vetter et al., 1993). For cytoarchitectural reference, every fourth section was counterstained with Cresyl Violet. To visualize the FG tracer, tissue sections were cover-slipped with anti-fading medium (ProLong, Molecular Probes) and viewed under ultraviolet light. Sections were photographed at high resolution with a Zeiss Axioskop 40 microscope using a Zeiss AxioCam MRc 5 digital camera (Zeiss, Oberkochen, Germany). High magnification micrographs of the labeled neuronal elements were obtained by photographing the same section at various planes of focus with a 40x (N.A. = 0.75) objective lens or a 100x (N.A. = 1.40) objective lens, stacking the images, and finally collapsing them into one single, maximum focus image using Helicon Focus Pro software (HeliconSoft Ltd., Kharkov, Ukraine). The brightness and contrast of images were adjusted with Adobe Photoshop software, version 17 (Adobe Systems Inc., San Jose, CA), and the illustrations were arranged into plates using Adobe Illustrator software, version 20 (Adobe Systems Inc.).

### Immunohistochemistry

Young adult mice of both sexes (*n* = 6; P18-24; body weight 22–25 g) were deeply anesthetized with sodium-pentobarbital and transcardially perfused with 0.9% NaCl followed by ice-cold 4% formaldehyde (prepared from freshly depolymerized paraformaldehyde) in 0.1 M phosphate buffered saline (PBS). Brains were removed from the calvarium and post-fixed for 2–3 h followed by immersion in a solution of 30% sucrose in PBS at 4°C overnight. Brains were sectioned into 30 μm thick transverse sections with a cryostat (Leica CM3050, Wetzlar, Germany) and collected in PBS. Sections were pre-incubated in 5–10% normal donkey serum (Jackson ImunoResearch Laboratories, West Grove, PA) in a blocking solution that contained 1% bovine albumin serum and 0.3% Triton X-100 in PBS for 1 h at room temperature. Sections were then incubated overnight at 4°C with the primary antibodies [goat anti-calretinin (AB1550; 1:500; Millipore, Solna, Sweden) and rabbit anti-KCC2 (potassium chloride cotransporter 2) (ANT-072; 1:200; Alomone Labs, Jerusalem, Israel)] diluted in a blocking solution that contained 2% normal donkey serum. On the following day, sections were washed three times with PBS and then incubated in darkness with the secondary antibodies Cy3-conjugated donkey anti-goat and Cy2-conjugated donkey anti-rabbit (Dianova, Hamburg, Germany) in blocking solution for 2 h at room temperature. Sections were then washed with PBS, mounted on gelatin-coated slides, cover-slipped with ProLong mounting medium, and stored in the dark at −20°C until visualization. The specificity of the immunoreactions was confirmed by pre-adsorbing each primary antiserum with the corresponding immunopeptide in excess, which led to the loss of immunoreactivity (data not shown). Immunolabeling was visualized with a laser scanning confocal microscope (Zeiss LSM510) equipped with Plan-Apochromat 63 ×/1.4 and 100 ×/1.4 DIC oil immersion objectives. All images were acquired and processed with AxioVision software (v. 4.8, Zeiss). The brightness and contrast of images were adjusted with Adobe Photoshop software, version 17 (Adobe), and the figures were arranged into a plate using Adobe Illustrator software, version 20 (Adobe).

### Recording procedures

The brainstem of pentobarbital-anesthetized mice (*n* = 42; P5-22) was quickly removed and placed in ice-cold low-sodium, high-sucrose artificial cerebrospinal fluid (aCSF), which contained (in mM) 85 NaCl, 2.5 KCl, 1.25 NaH_2_PO_4_, 25 NaHCO_3_, 75 sucrose, 25 glucose, 0.5 CaCl_2_, and 4 MgCl_2_, and bubbled continuously with 95% O_2_/5% CO_2_. Transverse brain slices containing the superior olivary complex were cut using a Vibratome (150–200 μm; Leica VT1200) and incubated for 20–30 min in normal aCSF, which contained (in mM) 125 NaCl, 2.5 KCl, 1.25 NaH_2_PO_4_ 2, 26 NaHCO_3_, 25 glucose, 2 CaCl_2_, and 1 MgCl_2_. Slices were transferred to a recording chamber perfused (~3 ml/min) with normal aCSF oxygenated at 36°C using an inline heater (SH-27B, Warner Instruments, Hamden, CT). Recordings were obtained within 4–5 h of the brain slice preparation. The following pharmacological agents (Tocris, Pittsburgh, PA) were used to block sodium and Ih currents, as well as inhibitory neurotransmission: tetrodotoxin (TTX; 1 μM), ZD7288 (20 μM), strychnine (0.5 μM), and SR95531 (5 μM). Drugs were dissolved in distilled H_2_O (10 mM), stored at −20°C, diluted, and added to the aCSF during the experiment.

Whole cell voltage clamp recordings were conducted on SPON and LSO neurons that were identified by their distinct locations in the brain slice and by their morphology (Helfert and Schwartz, 1987; Rietzel and Friauf, 1998; Saldaña and Berrebi, 2000). Recorded neurons were viewed with an upright microscope (Zeiss Axioscope) equipped with a digital charge-coupled device camera (Hamamatsu Orca2) using a × 40 water-immersion objective (Zeiss Achroplan) and infrared differential interference optics. Voltage clamp recordings were conducted with an amplifier (Molecular Devices Multiclamp700B, Sunnyvale, CA) using borosilicate glass microelectrodes (Harvard Instruments, Holliston, MA) with a final tip resistance of 2–8 MΩ. The internal pipette solution contained (in mM) 130 CsMeSO_4_, 5 NaCl, 10 HEPES, 1 EGTA, 1 CaCl_2_, 2 Mg-ATP, 0.3 Na_3_-GTP, 10 Na_2_-phosphocreatinine, adjusted to pH 7.3 with KOH. The series resistance was compensated by 70–80% and monitored throughout the experiment, and recordings where changes were >10% were discarded. Voltages were not corrected for the liquid junction potential. Neuron size was estimated from the capacitance compensation measurement and only neurons with a capacitance >20 pF were included in the analysis. Recorded signals were filtered with a low-pass four-pole Bessel filter at 10 kHz, sampled at 20 kHz, and digitized using a data acquisition interface (Digidata 1422A, Molecular Devices). Frequent fast miniature excitatory postsynaptic currents (mEPSCs) were recorded at a holding potential of −60 mV without stimulating the synaptic inputs. Glutamatergic synaptic events were pharmacologically isolated using a cocktail of TTX, strychnine and SR 95531 to block respectively ionotropic sodium, glycine, and GABA currents. In addition, ZD7288 was used to block Ih currents and thereby improve the voltage clamp of the SPON neurons and LSO principal neurons, which were recorded under the same conditions. The non-principal lateral olivocochlear (LOC) LSO neurons were first identified in current clamp based on resting membrane potentials that were more negative compared to principal neurons and tonic spiking during depolarizing current injection. LOC neurons are electrotonically very compact due to their small size (<20 pF) and lack of Ih (Fujino et al., 1997; Leijon and Magnusson, 2014) and, thus, can be voltage clamped easily without blockers.

### Extraction of mEPSC events

**Figure 1:**
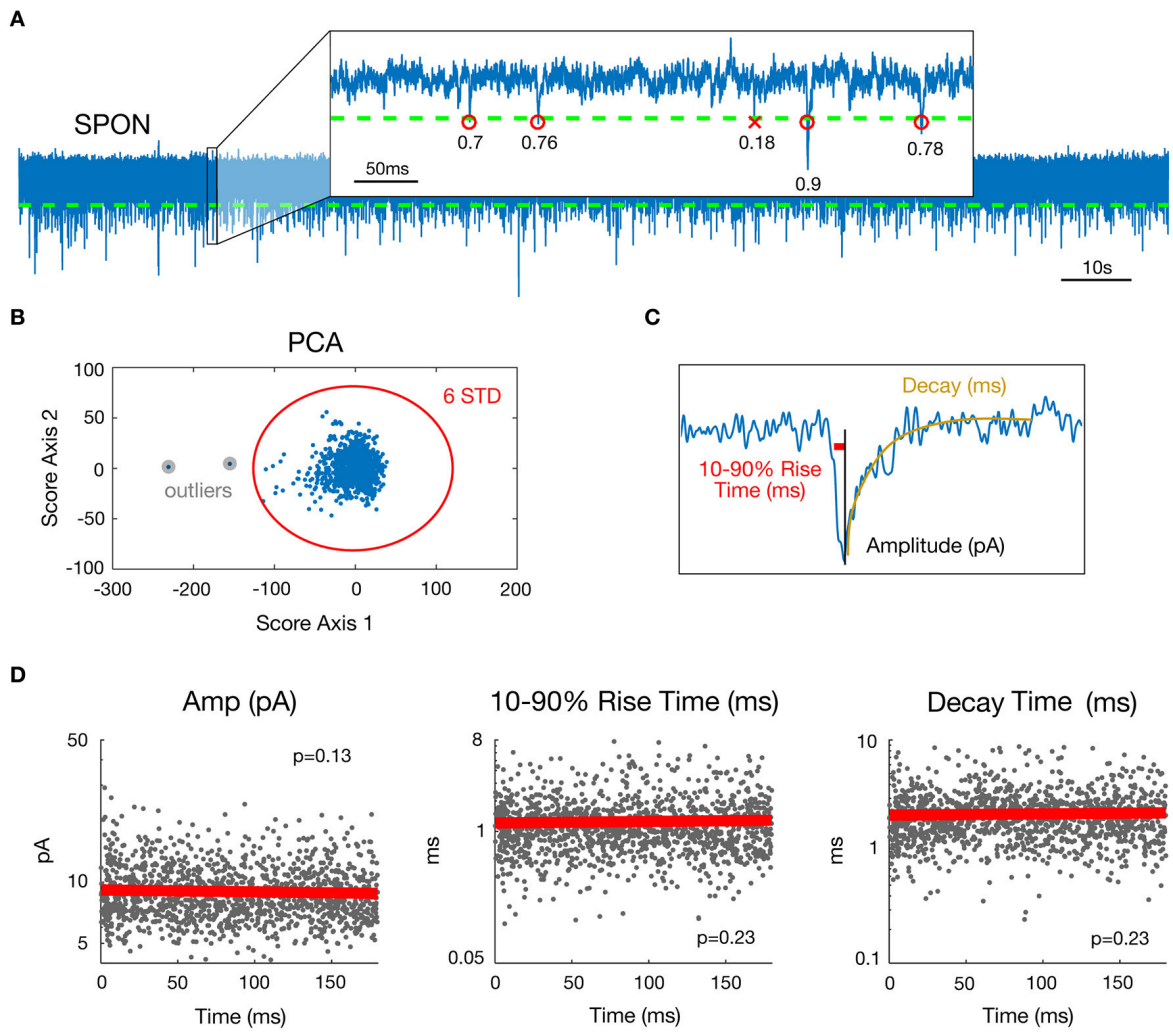
Estimation of mEPSC parameters in a representative SPON cell exhibiting spontaneous inward currents. **(A)** 180 s whole-cell recording time signal (top) and expanded 0.5 s time interval. Detection threshold for mEPSCs is represented by a dashed line and R^2^ goodness-of-fit coefficients for the linear exponential mEPSC model are indicated for each detected event. **(B)** As a step of outlier detection, a principal component analysis was applied to the matrix of mEPSC time signals. Events that appeared beyond the mean ± 6 SD of at least one of the two first components were removed then process was iterated (one time was enough in the example shown here). **(C)** Estimated parameters illustrated for one detected mEPSC. The Decay parameter corresponds to the time constant of the exponential decay shown in yellow. **(D)** Stability over time assessment for the three mEPSC parameters estimated from the model. The plain bold line is the linear regression and all *p*-values for the *F*-test were >0.0037, defined as the significance threshold after Bonferroni correction (see methods).

Preliminary to mEPSC detection, data were de-trended by high-pass filtering (Butterworth, *fc* = 5 Hz) and de-noised by low-pass filtering (Butterworth, *fc* = 4 kHz). A cascade of Butterworth bandstop filters [*fc* = n^*^50+[−1 1] Hz, *n* = 1–80] removed 50 Hz interference and related harmonics up to 4 kHz. To detect miniature events (each including one mEPSC), a threshold was set for the data at mean minus four standard deviations (SD) (Figure 1A). Possible artifact influence on mean and SD estimates was lowered by working only on data ranging between percentiles 1% and 99%. Events following a first event by <3 ms were removed, as their parameter estimates may be biased by the decay of the first event.

To estimate the individual mEPSC parameters, the nlinfit function in Matlab (The Mathworks®) for nonlinear least squares fitting of the mEPSC was used, which applies the Levenberg-Marquardt algorithm (Seber and Wild, 2003). Since a double exponential fit (Roth and van Rossum, 2009) for mEPSCs led to multiple local minima, i.e., several possible values for decay and rise time parameters as well as erroneous amplitude estimates, a more robust mEPSC model involving a linear rise component and an exponential decay (Jonas et al., 1993) was employed (Figure 1C). The mEPSC was fitted with the following function:

$$EPSC\left(t\right)=\{\begin{array}{c} \begin{array}{l} Amplitude\left(\frac{t-t_{0}+Rise}{Rise}\right)if\;t_{0}-Rise<t\\ \;\leq t_{0}\;\left(rising\;part\right) \end{array}\\ Amplitude\left(e^{-\frac{t-t_{0}}{Decay}}\right)\;if\;t>t_{0}\;\left(decay\;part\right) \end{array}$$

where *Amplitude, Rise, t*_0_, and *Decay* are the four parameters of the model and where *t*_0_ is the time of the maximum amplitude of the mEPSC.

For each mEPSC, the goodness of fit was then evaluated by the coefficient of determination R^2^ (Glantz and Slinker, 1990). To ensure that only correctly fitted and biologically plausible events were included, the following rules were applied to the data: {*R*^2^ > 0.3; 0.1 < *Decay* <15 ms; 0.05 < *10–90% rise time* <10 ms; *Amplitude* <200 pA}. This accounts for 72.9% of all detected mini-Events. A principal component analysis (Jolliffe, 2002) was applied to the matrix of mEPSC time signals (one mini-Event is an observation and time values are seen as variables) as a final step of outlier detection (Figure 1B). Events that appeared beyond the mean ± 6 SD of at least one of the two first components were removed. This process was iterated on the matrix of remaining mini-Events until no new mini-Event was selected. This step removed 0.14% of events by cell on average (min 0%; max 2.27%). Removing outliers was important for the distribution and multi-dimensional analysis of the dataset. The outlying events were, however, so rare and heterogeneous that they cannot reasonably correspond to a putative input. Finally, visual inspection was done for all cells to ensure that no mini-Events were missed and that no artifacts were detected as mini-Events. The final three parameters selected in this database for analysis were peak amplitude, 10–90% rise time, and decay time.

### Statistical analysis of mEPSC parameters

For each cell, stationarity over time of the three chosen parameter values was assessed by linear regressing values against time (Figure 1D). For a given parameter and a given cell, stationarity was considered as acceptable if the regression model was considered as null by the *F*-test. The significance threshold for the *p*-value here was 5%/3/45 = 0.37% after Bonferroni correction for multiple tests performed across the 3 parameters and 45 cells available. Only neurons for which mEPSC parameters were stationary over time were selected (SPON, *n* = 19; LSO, *n* = 13; LOC, *n* = 13). Non-stationary parts of some recordings (10 s up to 120 s at most for six cells) were visually-detected and removed.

Since parameter probability distributions were skewed and close to a log-normal distribution for all cells, we systematically displayed them using a log scale for parameter values and we used the median instead of the mean to characterize the distribution central value. Pearson's linear correlation coefficient and its *p*-value based on Student's t distribution for a transformation of the correlation (Rahman, 1968) were used to analyze the set of mean values in **Figure 5**. The *p*-value threshold was 5%/9 = 0.56% after Bonferroni correction for the 9 tests performed in **Figure 5**.

### Cluster analysis

The existence of several distinct types of mEPSCs within recordings of a given cell was assessed by clustering parameter pair values {Log(peak amplitude); Log(decay time)}. We applied a 2D clustering algorithm that finds local maxima in the density of the extracted data point and separates them as peaks in clouds (Rodriguez and Laio, 2014). The main idea of such clustering is that cluster centers are characterized by a higher density than their neighbors and by a relatively large distance from points with higher densities. This method automatically estimates the number of clusters and only uses one free parameter, the cutoff distance *dc*, to estimate the local density of a point. According to Rodriquez and Laio, on a large dataset, the *dc* choice should not have any influence on the clustering results and is typically chosen as the percentile 1 or 2% of the total distance dataset. To minimize the risk of missing a cluster due to the *dc* value, we chose the clustering results where the maximum number of clusters was found among four *dc* values between 0.5 and 4%.

## Results

### Neuronal tract tracing clarifies inputs from the cochlear nucleus to the SPON

**Figure 2:**
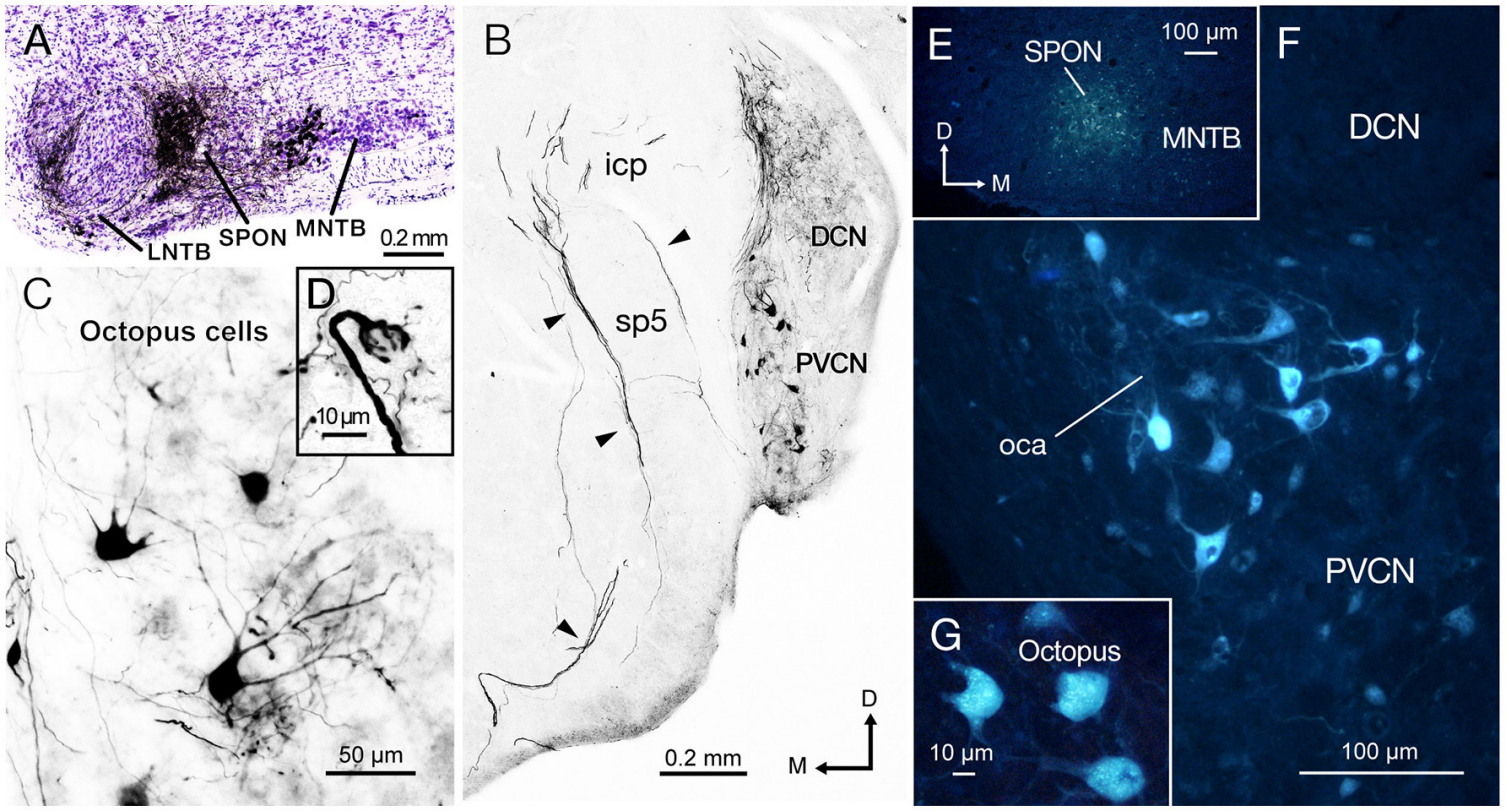
The injection of biotinylated dextran amine (BDA) and Fluoro-Gold (FG) into the superior paraolivary nucleus (SPON) reveal a prominent projection of octopus cells from contralateral posteroventral cochlear nucleus (PVCN). **(A)** Micrograph of Nissl-counterstained coronal section through the center of the BDA injection site. **(B)** Low-magnification view of the PVCN contralateral to the injection site, which contains retrogradely labeled octopus cells. The injection of BDA into the SPON labels efficiently also the axons of octopus cells through the intermediate acoustic stria (IAS; arrowheads). **(C)** High magnification of typical labeled octopus cells. **(D)** Detail of a section showing the calycial nerve endings of octopus cell axons that innervate the ventral nucleus of the lateral lemniscus (VNLL) ipsilateral to the injection site and contralateral to the parent cell body. **(E)** Micrograph of a coronal section through the center of the FG injection site. The deposit has a low degree of spread but the tracer remains within the boundaries of the nucleus. **(F)** The contralateral PVCN exhibits abundant retrogradely labeled cells bodies in the octopus cell area (oca). **(G)** High magnification of the labeled octopus cells following single injection of FG into the SPON. Orientation arrows in **(B)** apply also to (**C,F**, and **G**). Orientation arrows in E apply also to **(A,D)**.

We evaluated mice with a single discrete injection of BDA confined to the SPON (Figure 2A). The presence of labeled thick fibers that curve around the inferior cerebellar peduncle and circumvent the spinal tract of the trigeminal nerve is compatible with the intermediate acoustic stria (IAS; Figure 2B; Smith et al., 2005). Retrogradely labeled neurons were found in the PVCN with a clear contralateral predominance (Figure 2B), and they were readily identified as octopus cells based on their distinct dendritic shape (Figure 2C; Harrison and Irving, 1966; Saldaña et al., 1987; Pocsai et al., 2007; Bazwinsky et al., 2008). These cells had large, irregularly shaped cell bodies (~ 20 μm in diameter) with three to five thick primary dendrites emerging from only one side of the cell body (Figure 2C). The same experiments revealed labeled calyx-like endings in the ventral nucleus of the lateral lemniscus (VNLL) ipsilateral to the injection site (Figure 2D inset) (and hence contralateral to most labeled octopus cells), thus strengthening the conclusion that our injections had efficiently labeled octopus cells and their projections (see also Vater and Feng, 1990; Adams, 1997; Schofield and Cant, 1997). Retrogradely labeled neurons were found also in inhibitory structures known to project to the SPON, including the medial and lateral nuclei of the trapezoid body (MNTB and LNTB) (Figure 2A; see also Saldaña et al., 2009; Viñuela et al., 2011), as well as in the ventral tectal longitudinal column (TLCv; not shown), which may contain a mixed population of GABAergic and glutamatergic neurons (Aparicio and Saldaña, 2014).

To verify the results obtained with BDA and to confirm the observed connectivity at younger ages, we injected the retrograde tracer (FG) in the SPON of three additional animals. When the FG was deposited within the borders of SPON (Figure 2E), robust labeling of the octopus cell area (oca) in the contralateral PVCN (Figure 2F) was observed, whilst no labeling in either the ipsilateral PVCN or the AVCN on both sides was seen. Once more, the large irregular shaped neurons labeled in the contralateral PVCN were identified as octopus cells (Figure 2G).

### Calretinin-immunolabeling demonstrates that the IAS densely innervates the SPON

Calretinin immunolabeling has been shown to label the octopus and bushy cells of the cat, including their axons (Adams, 1997). Therefore, we performed immunostaining for calretinin to visualize the IAS from the PVCN to the superior olivary complex (Cant and Benson, 2003), and followed the labeled axons to the SPON.

**Figure 3:**
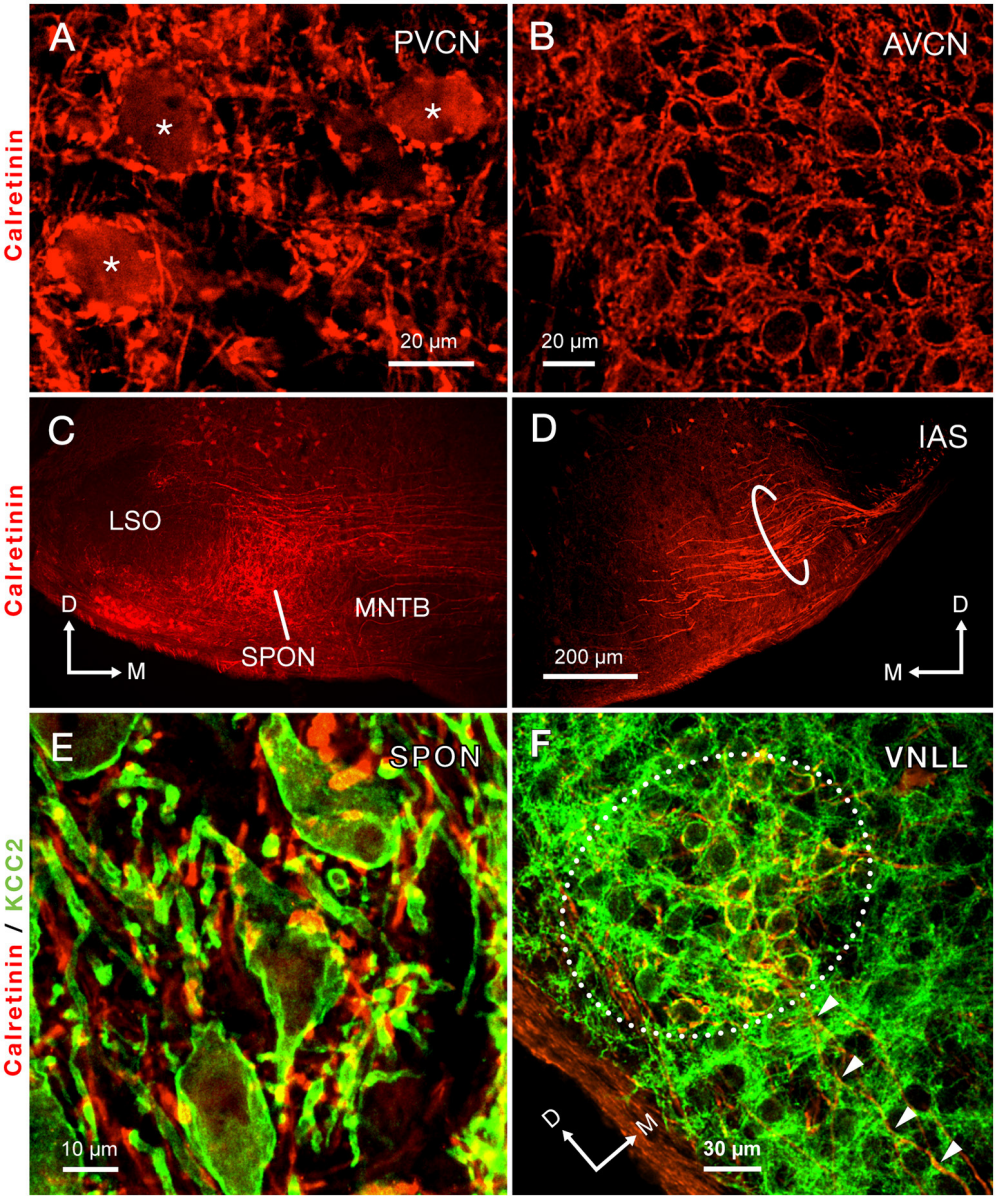
Immunolabeling of calretinin reveals the trajectory of the intermediate acoustic stria (IAS) from the posteroventral cochlear nucleus (PVCN) to the contralateral superior olivary complex. **(A)** Micrograph depicting the cell bodies of octopus cells densely immunolabeled for calretinin (asterisks). **(B)** The cell bodies of neurons of the anteroventral cochlear nucleus (AVCN) are immunonegative for calretinin and are surrounded by abundant calretinin-positive terminals. **(C)** A dense plexus of calretinin-positive fibers that presumably originate from the contralateral IAS innervates the superior paraolivary nucleus (SPON). Notice that areas bordering the SPON that correspond to the medial nucleus of the trapezoid body (MNTB) and the lateral superior olive (LSO) are largely devoid of labeled fibers. **(D)** Micrograph of the ventral portion of the IAS, as it approaches its ipsilateral SOC. The thick, calretinin-positive axons presumably belong to octopus cells. **(E)** Co-staining of calretinin-positive fibers (red) and the postsynaptic marker potassium-chloride cotransporter 2 (KCC2; green) indicates that the SPON is densely innervated by calretinin-positive fibers, which presumably belong to octopus cells. **(F)** Co-staining of calretinin and KCC2 also demonstrates large calretinin-immunolabeled calyx-like synaptic specializations, which presumably belong to octopus cells, surrounding the cell bodies of neurons of the ventral nucleus of the lateral lemniscus (VNLL). The arrowheads indicate calretinin-positive axons approaching the VNLL. Orientation arrows in D also apply to **(A,B)**. Orientation arrows in **(C)** also apply to **(E)**. Calibration bar in **(D)** also applies to **(C)**.

Calretinin densely labeled the auditory nerve fibers and, thus, was present throughout the ventral CN (Figures 3A,B). The densest calretinin-staining was observed in the PVCN (Figure 3A), particularly in the octopus cell area (Osen, 1969; Lohmann and Friauf, 1996). In this region, large immunopositive cell bodies, identified as octopus cells, were delineated by calretinin-labeled nerve endings (Figure 3A). In contrast, bushy cells of the AVCN received pre-synaptic calretinin-positive terminals, but were devoid of calretinin labeling within their cell bodies (Figure 3B).

The morphology and trajectory of the axons immunolabeled in the IAS matched the tract tracing results shown in Figure 2. Upon reaching the ventral brainstem, calretinin-labeled fibers of the IAS spread out to bypass the LSO dorsocaudally (Figure 3D). As the IAS crossed the midline, it bypassed the MNTB dorsally without innervating it. In contrast, abundant collaterals terminated in the SPON as an intricate network of thick diameter calretinin-positive fibers (Figure 3C). The LSO was devoid of calretinin staining (Figure 3C), consistent with a well-established absence of IAS input (Cant and Benson, 2003). Counterstaining of the SPON with the neuron-specific potassium chloride co-transporter (KCC2; Blaesse et al., 2006; Kopp-Scheinpflug et al., 2011) provided labeling of the postsynaptic membrane. Dense co-labeling of calretinin-KCC2 puncta on cell bodies and dendrites indicated that SPON neurons receive rich innervation from thick calretinin fibers (Figure 3E). Beyond the superior olivary complex, the immunolabeled fibers coursed rostrolaterally to terminate in the VNLL, forming calyx-like endings (Figure 3F). The presence of calretinin-labeled terminal fibers in both the VNLL and SPON supported the notion that calretinin-positive fibers and puncta in the SPON originated from the PVCN (Vater and Feng, 1990; Adams, 1997; Schofield and Cant, 1997). These results strongly suggest that the octopus cells of the mouse provide substantial input to both the SPON and the VNLL (see also Adams, 1997; Schofield and Cant, 1997). In contrast to the IAS, the ventral acoustic stria (VAS), which contains fibers of bushy and planar multipolar cells of the CN and also provides substantial input to the contralateral superior olivary complex, was not labeled by calretinin immunostaining in the mouse.

### mEPSC analysis indicates one predominant excitatory input to the majority of SPON neurons

In the brain, one type of synaptic input onto a neuron results in shared postsynaptic properties. Conversely, inputs that originate from multiple types of presynaptic inputs typically produce heterogeneous properties of the postsynaptic neuron (Branco and Staras, 2009). Miniature excitatory postsynaptic currents (mEPSCs) are synaptic events that consist of discrete units (quanta; del Castillo and Katz, 1954) that occur with a certain amplitude and probability. The stochastic nature of neurotransmitter release from nerve terminals (Ribrault et al., 2011), even if the incoming fibers lack connection with their cell bodies, enables the recording of excitation in the form of mEPSCs from all possible sources of synaptic terminals impinging on a given neuron. We reasoned that, if the mEPSC events recorded in the SPON originate from multiple cell types, it should be reflected in their amplitude, kinetics or frequency distribution.

To examine whether SPON neurons receive multiple types of excitatory inputs, we compared mEPSC parameters with those from mEPSCs recorded from the neighboring LSO. Like the SPON neurons, principal neurons of the LSO are excited by a few strong fibers per neuron at the age range studied here (Case et al., 2011; Felix and Magnusson, 2016; Lee et al., 2016) and are thought to arise from one predominant input from spherical bushy cells of the AVCN (Cant and Casseday, 1986; Cant and Benson, 2003). However, this view is complicated by the fact that in addition to the spherical bushy cells, the LSO also receives excitatory inputs from planar multipolar cells (Gómez-Álvarez and Saldaña, 2016). We hypothesized that the planar multipolar cells may preferentially target the non-principal LOC LSO neurons (Campbell and Henson, 1988; Brown and Levine, 2008). To clarify this circuitry, we tested the hypothesis that the non-principal LOC neurons, known to express specific intrinsic membrane properties that make them suitable for slow integration of synaptic inputs (Fujino et al., 1997; Leijon and Magnusson, 2014), receive two types of excitatory synaptic inputs, whereas principal LSO neurons receive one type of input, as previously suggested.

**Figure 4:**
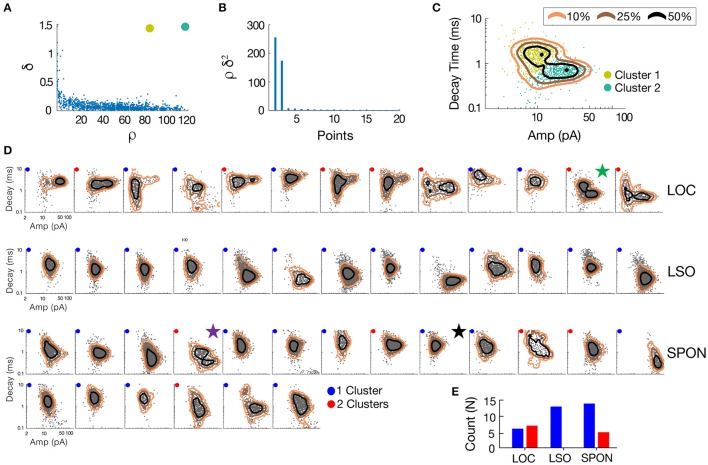
mEPSC parameter distribution shows evidence for one or two clusters of excitatory inputs. A simple but robust 2D clustering algorithm (Rodriguez and Laio, 2014) was applied to scatter plots of mEPSC amplitude vs. decay time. We illustrate the algorithm principle on an individual LOC example **(A)**. In brief, for each data point of the scatter plot, we compute its local density ρ and its euclidean distance δ from points of higher density **(A)**. The local density of a given point is equal to the number of points that are closer to this point than a cutoff distance (chosen typically as a percentile of distance between all pair of points, 2% here). Cluster centers (in yellow and green) are recognized as points for which both ρ and δ are simultaneously large. A score, such as ρ.δ^2^ computed for each point and sorted in descending order **(B)** helps identify the number of clusters present in the data. After the determination of cluster centers, each remaining point is assigned to the same cluster as its nearest neighbor of higher density **(C)**. The probability (10–50%) of the point distribution is highlighted by contour lines to improve visualization of the distribution peaks which should be and actually are consistent with identified clusters. **(D)** Scatter plots of mEPSC amplitude vs. decay time for the entire sample of LOC, LSO principal, and SPON neurons where contour lines are highlighted as in *C*. The algorithm produced one (blue circle) or two (red circle) cluster peaks. **(E)** A single cluster of mEPSCs was found for most SPON neurons (14/19) and for all LSO principal neurons (13/13), whereas more than half of the LOC neurons (7/13) had two clusters, suggesting two types of inputs. Plots marked by stars were chosen for closer scrutiny for evidence of multiple inputs (Figure 5).

**Figure 5:**
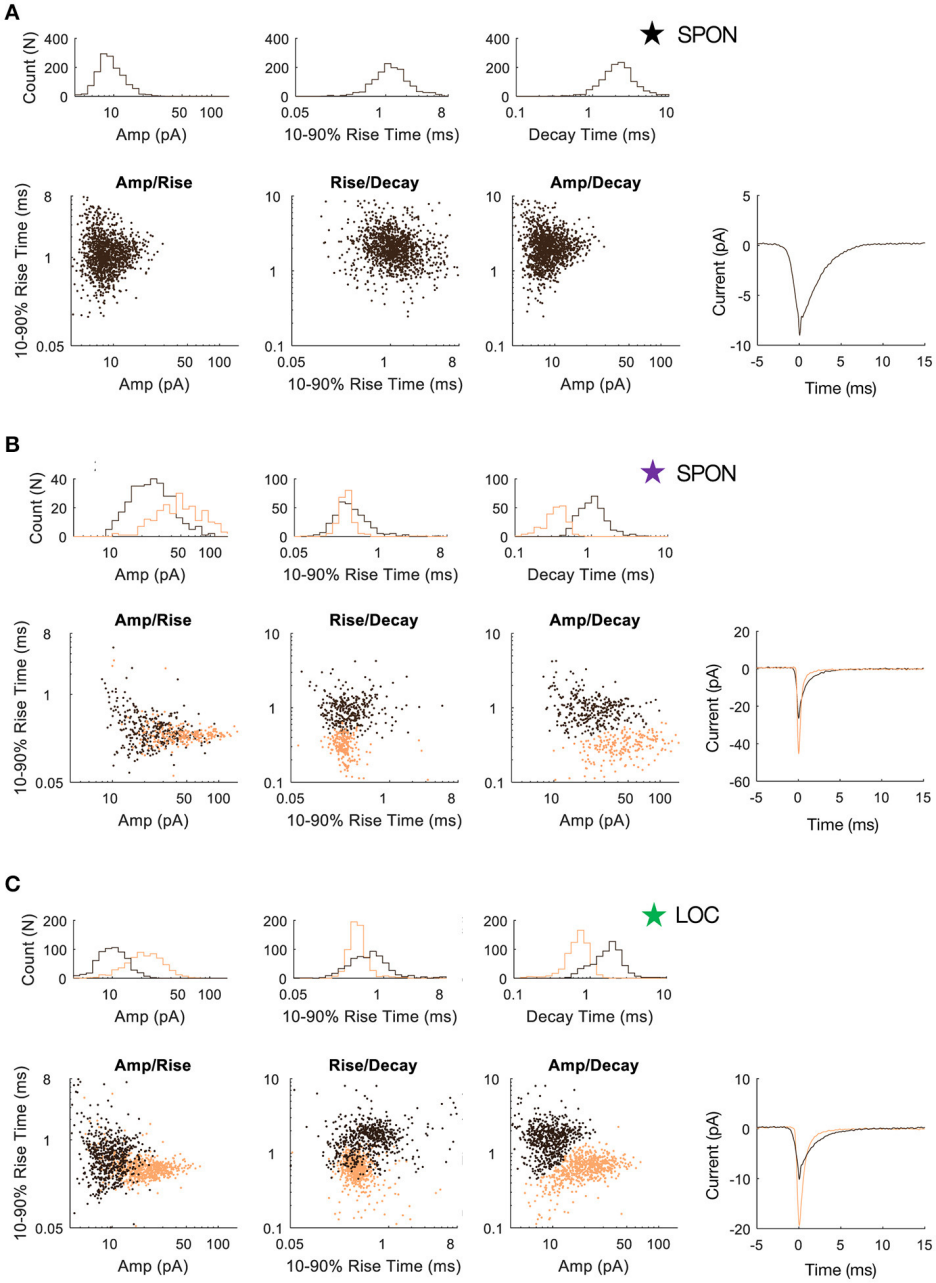
SPON neurons exhibit variability of mEPSC parameters. **(A)** Log distributions of peak amplitude, 10–90% rise time, and decay values (top) and scatter plots between parameters (bottom) for a representative SPON neuron with a homogeneous excitatory input. The average of all detected miniature EPSCs is shown in the lower right plot. **(B)** Log distribution and scatter plots for a SPON neuron that exhibited heterogeneous EPSC parameters. Two populations of EPSCs, identified from applying a 2D clustering method on the Amp/Decay raster plot, are depicted in black and orange in each plot. **(C)** For comparison, EPSC parameters for a non-principal lateral olivocochlear (LOC) LSO neuron are shown. Each example neuron is denoted with a star that corresponds to data shown for the same neurons in Figure 4.

To determine whether patterns in the mEPSC data reflected different types of synaptic inputs, we first applied a 2D clustering algorithm (Rodriguez and Laio, 2014) on plots of amplitude-decay parameters. The principle of this algorithm is to identify local maxima in the density of the extracted data points (Figure 4A) and therefore the number of clusters present in the data (Figure 4B) before assigning all points to one of the clusters. Applied to an LOC neuron, the algorithm found two clusters, the clouds of which are consistent with the two modes of the points' distribution (Figure 4C). This analysis provided the means to search for clusters with an arbitrary shape in an unbiased manner. Clustering results are shown as point colors in the amplitude-decay plots (the parameter pair which revealed the most patterns; Supplementary Figure 1) for populations of LOC, principal LSO, and SPON neurons (Figure 4D). A summary of clustering results (Figure 4E) shows that one cluster was found for most SPON neurons (14/19) and for all LSO principal neurons (13/13). In contrast, slightly over half of LOC neurons (7/13) had two clusters, indicating two types of inputs. Scatter plots and distribution histograms of parameter pairs for three neurons, two in the SPON (Figures 5A,B) and one LOC (Figure 5C), were selected from the examples in Figure 4 (denoted by corresponding stars) and scrutinized for evidence of multiple inputs. When there was one single cluster, such as the SPON example in Figure 5A, histograms of all recording parameters were unimodally distributed. However, when two clusters were present, such as the SPON example in Figure 5B, histograms of amplitude and decay time parameters corresponded to two distribution peaks. This type of bimodal pattern found in some SPON neurons resembles the distribution pattern found in the electrotonically compact LOC neurons (Fujino et al., 1997; Leijon and Magnusson, 2014) with two clusters (Figure 5C).

**Figure 6:**
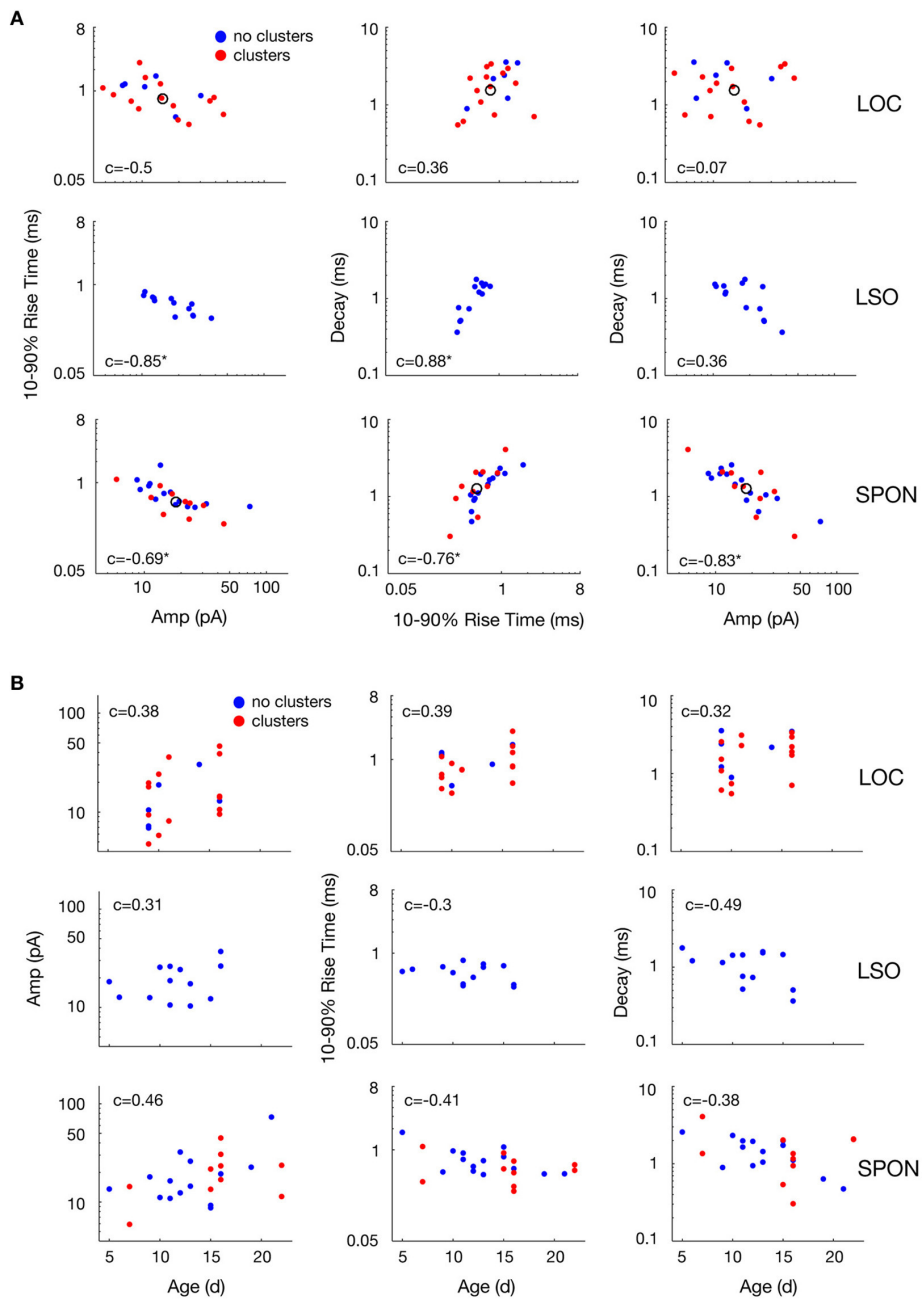
Variability of mean mEPSC parameter values of LOC, LSO, and SPON clusters. **(A)** Scatter plots between mean values of peak amplitude, 10–90% rise time, and decay parameters for all clusters revealed by the clustering analysis as in Figure 5 (red points) or cells showing no clusters (blue points). One dot corresponds to the mean of one cluster and the open circle depicts the median of all mean values. **(B)** Variability with age of mean mEPSC parameter values of LOC, LSO and SPON clusters. Scatter plots between median values of peak amplitude, 10–90% rise time, and decay time (left to right, in ordinate) for all clusters as in Figures 5A–C, 6A and animal's age (abscissa). Each column of plots corresponds to one parameter in the abscissa and each line of plots corresponds to another parameter in the ordinate. Spearman's correlation values between two parameters are indicated inside the plot. Correlation value is marked by an asterisk when significant (see Materials and Methods).

The heterogeneity within the clouds in the amplitude-decay scatter plots suggests more subtle variations of the input distributions or differences of the waveform parameters/shape. The latter possibility might be related to how the inputs are compartmentally distributed in a neuron (Gardner et al., 1999; Magee, 2000). To assess whether the excitatory inputs could be categorized based on SPON neuronal subtypes (Felix et al., 2013), i.e., that a neuron receives one type of excitatory input but that the type of input varies between cells, the mean mEPSC parameters were quantified for comparisons across the cell populations to seek evidence for patterns (Figure 6A). The respective cluster averages indicated no clear separation of the parameters in any of the cell types. The mEPSC amplitude was negatively correlated with both 10–90% rise time and decay of the mEPSCs in SPON and LSO principal neurons, but that type of correlation was absent in the LOC neurons. We also observed slightly larger intercell variability of mEPSCs parameters in SPON than in LSO neurons. The clusters were also plotted against the age of the animal, which, however, did not reveal any correlation (Figure 6B).

## Discussion

This study presents a combination of anatomical and physiological evidence for one predominant excitatory input from the octopus cells in the PVCN to most neurons of the contralateral SPON. This result challenges the view that SPON neurons receive multiple prominent excitatory inputs from the CN (Zook and Casseday, 1985; Friauf and Ostwald, 1988; Thompson and Thompson, 1991; Schofield, 1995; Saldaña et al., 2009). A strong excitatory input from the octopus cells, known to encode broadband temporal acoustic information (Rhode, 1998; Oertel et al., 2000), is consistent with the highly synchronous sound-evoked onset spiking response described in the SPON (Grothe, 1994; Kuwada and Batra, 1999; Behrend et al., 2002; Dehmel et al., 2002; Felix et al., 2013). This onset response coupled with precise offset spiking driven by a rebound from MNTB-derived inhibition (Felix et al., 2011; Kopp-Scheinpflug et al., 2011), enables the SPON to segment vocalized sounds using information from two of the most temporally secure acoustic pathways in the brain.

### Technical considerations for tract tracing and immunolabeling experiments

The main focus of this study was to investigate the excitatory projections from the CN to the SPON and thus, details about other potential sources of inputs highlighted by the tracer were not examined. These tracer injections reproduced the main inhibitory inputs to the SPON reported in the rat (Saldaña et al., 2009; Viñuela et al., 2011) that resulted in robust retrograde labeling of the glycinergic inputs (Magnusson et al., 2005; Roberts et al., 2014) from the ipsilateral MNTB and LNTB (Banks and Smith, 1992; Sommer et al., 1993; Saldaña et al., 2009). Likewise, the tracers labeled the neurons of the more recently discovered midbrain structure TLCv (Saldaña et al., 2007; Viñuela et al., 2011). Taken together, these findings demonstrate that the mouse and rat share a similar organization of these pathways. It is noteworthy that we did not observe any reciprocal labeling in the medial superior olive (MSO), indicating that the mouse differs from the gerbil, in which the MSO has been reported to both receive projections from, and project to, the SPON (Kuwabara and Zook, 1999; Stange et al., 2013). Rather, the connectivity of the SPON and MSO in the mouse resembles the organization described for the rat (Saldaña et al., 2009).

When injecting BDA extracellularly, one must always consider the possibility that the tracer may spread outside the area of interest. If, for instance, the lateral areas of the MNTB were contaminated by the injection site, the tracer would presumably be taken up by terminals of globular bushy cells of the contralateral AVCN that project to the MNTB (Kuwabara et al., 1991). Another possibility of tracer uptake is via *en passant* fibers that cross the injection site, such as the axons of the spherical bushy cells that innervate the contralateral MSO (Cant and Casseday, 1986) or the multipolar cells in the PVCN projecting to the contralateral ventral nucleus of the trapezoid body (Darrow et al., 2012). Thus, the precision of the injection site is crucial when investigating the origins of the inputs to these small brainstem areas. When comparing the inevitable variability between the different cases of tracer injections, it became clear that a very discrete deposit in the SPON almost exclusively resulted in retrograde labeling of octopus neurons in the contralateral PVCN (Figure 2E). The fact that tracer deposits spreading to adjacent areas gave rise to varying labeling of the ipsilateral PVCN and multiple cell types throughout CN, could explain the difference between the present and previous tracing studies in the SPON (Zook and Casseday, 1985; Friauf and Ostwald, 1988; Thompson and Thompson, 1991; Schofield, 1995; Saldaña et al., 2009).

The tracer experiments clearly demonstrated that the octopus cells of the PVCN provide a major input to the contralateral SPON. However, the fibers labeled by BDA did not allow us to evaluate the potential strength of this IAS-projection. Recently, an excitatory synaptic input, speculated to be of an octopus cell origin based on its physiological properties, was documented in SPON brain slices (Felix and Magnusson, 2016). The same study compared the strength of evoked synaptic excitation in SPON to excitation triggered in the adjacent LSO, and found them to be of similar magnitude (Felix and Magnusson, 2016). The calretinin immunolabeling, which enabled visualization of how the IAS spreads in the ventral auditory brainstem supports the conclusion that the octopus cell projection to the contralateral SPON is of considerable strength, and presumably represents the neural substrate for the onset response to sounds recorded from the SPON *in vivo* (Grothe, 1994; Kuwada and Batra, 1999; Behrend et al., 2002; Dehmel et al., 2002; Felix et al., 2013). The lack of calretinin-labeled fiber endings in the LSO and MNTB suggests that the VAS does not provide a substantial input to the SPON. However, we cannot exclude that some immunolabeled fibers originated from adjacent LNTB neurons (Figure 2A; see also Saldaña et al., 2009) that were highlighted by the calretinin stain.

### Technical considerations for mEPSC clustering

Our clustering of mEPSCs is similar in its essence, purpose, and weaknesses to what is classically done in spike-sorting analysis, for instance (Rey et al., 2015). There is no widely used absolute method for such a purpose, but the two typical steps are to obtain a cloud of points (e.g., mEPSCs) that visually emphasizes patterns, and then choose a clustering method which gives reasonable mathematical results along with a convincing fit from visual inspection. From the linear-exponential fitting model used for mEPSC events (see methods), we were able to extract three parameters: 10–90% rising time, amplitude, and decay time, which allowed clustering in those three dimensions of data or using a two dimensional subset of parameters. Subsequently, many classical clustering methods were applied to the data including k-means, Gaussian mixtures, and silhouette estimates, but they were not consistent with visual inspection of clear individual examples, such as Figure 4A. Instead, a recent clustering method (Rodriguez and Laio, 2014) that is restricted to two dimensional data eventually led to the most convincing results, by far, in terms of the fit with visual inspection, as well as more quantitative computations, such as distribution peaks (Figure 4). Since clustering cannot be objectively validated on non-simulated data, we chose to show the clustering results for all available cells along with distribution peaks (Figure 4). While the categorization of a couple of cells could always be debated, this clustering method intuitively follows distribution peaks of data as long as those latter ones constitute a significant percentage of data (i.e., a cluster cannot build on a few percent of the data). However, the restriction of this clustering method to 2D data imposes a choice on a mEPSC parameter pair. Finally, we explicitly disadvantaged the hypothesis of a single input (cluster) on data by systematically testing several thresholds—the only free parameter of the clustering method—for each cell and picking up the largest number of clusters found. One must always must keep in mind however that our method relies on the hypothesis that two distinct inputs would lead to separate mEPSC parameters. Even if this is highly unlikely, if for all cells, two distinct inputs to SPON cells had absolutely similar properties in a systematic fashion, they would obviously remain invisible to our method.

### mEPSC variability is not related to the recording condition

It is conceivable that the voltage clamp recordings make the data prone to space clamp errors (Bar-Yehuda and Korngreen, 2008). Imperfect voltage control of the cell reduces the driving force, i.e., the difference in the voltage of the clamped neuron and the reversal potential of the excitatory currents, in the distal dendrites compared to in the cell body where the recording occurs (Spruston et al., 1993). Consequently, mEPSCs that originate far out on the dendrites will be attenuated as a function of distance from the cell body. Inclusion of the electrotonically compact LOC neurons (Fujino et al., 1997; Leijon and Magnusson, 2014) thus served as an internal control for the detection of differential excitatory inputs, as they would be much less affected by space clamp errors than both the large multipolar SPON neurons (Saldaña and Berrebi, 2000) and the large bipolar LSO principal neurons (Helfert and Schwartz, 1987; Kulesza, 2007). The fact that the mEPSC amplitude was negatively correlated with both 10–90% rise time and decay for SPON and LSO principal neurons, but that such correlation was absent in the LOC neurons, strengthens the hypothesis that the aforementioned excitatory inputs may be subjected to dendritic filtering (Gardner et al., 1999; Magee, 2000). Additionally, the slightly larger intercellular variability of mEPSC parameters in SPON compared to LSO neurons may relate to the input-distribution with respect to the neurons' geometric shape (SPON: Saldaña and Berrebi, 2000; Felix et al., 2013, LSO: Helfert and Schwartz, 1987; Kulesza, 2007). However, the fact that two clusters of parameter values were clearly evident for some SPON neurons demonstrates that space clamp errors were not precluding the detection of mEPSCs with different kinetics and amplitudes in these recordings. When we take into account that SPON neurons are rather homogenous in size and dendritic structure (Saldaña and Berrebi, 2000; Felix et al., 2013), similar to LSO principal neurons (Helfert and Schwartz, 1987), and have compact mEPSC properties (Felix and Magnusson, 2016), we conclude that the majority of the SPON neurons predominantly receive a single type of excitatory input.

### Pre- and postsynaptic properties are input specific and can distinguish multiple sources of inputs

The mEPSCs recorded in this study are action potential-independent (Ramirez and Kavalali, 2011) and reflect stochastic release of neurotransmitter (quanta) from all synaptic terminals impinging on the neuron (del Castillo and Katz, 1954; Ribrault et al., 2011). The quantal release of neurotransmitter is unique to each synapse type (Branco and Staras, 2009), thus two functionally separate inputs are expected to manifest as variations in quantal properties, such as their size. Two functionally separate inputs should consequently result in two populations of mEPSC amplitudes (Borst et al., 1994; Granseth and Lindström, 2003), which indeed was the case for the majority of the LOC neurons and for some SPON neurons. Also, postsynaptic properties, such as the density and subunit composition of postsynaptic AMPA receptors, could contribute to the segregation of mEPSCs from different fiber types according to mEPSC amplitude and decay times (Takahashi et al., 1995; Masugi-Tokita et al., 2007). For example, in the dorsal CN, the mEPSCs of the fusiform cells display clear bimodal distribution reflecting two sources of excitatory inputs (Gardner et al., 1999). The fast mEPSCs are from presumptive auditory nerve fibers (Gardner et al., 1999) since these inputs mainly interact with GluR4 subunits (Rubio and Wenthold, 1997) with fast kinetic properties (Mosbacher et al., 1994). Conversely, slower mEPSCs are presumed to originate from parallel fibers (Gardner et al., 1999), which may interact with AMPA receptors with slow calcium-impermeable GluR2 subunits (Gardner et al., 2001). In this study, the amplitude-decay relationship indeed proved to be the most useful property for assessing the distribution patterns, demonstrating clear separation of mEPSC populations in both LOC and SPON neurons.

The origins of the two presumptive types of inputs to the LOC is presently unknown, but it was recently demonstrated by anatomical tract tracing that 44% of the brainstem neurons that innervate the rat LSO are excitatory spherical bushy cells, and 13% are excitatory planar multipolar cells (Gómez-Álvarez and Saldaña, 2016). Based on the clustering of mEPSCs in the LSO, our data suggest that the principal neurons receive a homogenous class of excitatory input, presumably from spherical bushy cells (Cant and Casseday, 1986; Cant and Benson, 2003), and that the majority of non-principal LOC neurons receive heterogeneous input, possibly from both spherical bushy cells and planar multipolar cells or a combination with an unknown source, such as an input from descending pathways (Schofield and Cant, 1999; Doucet et al., 2002; Saldaña, 2015). A single excitatory input to LSO principal neurons is also supported by the low convergence ratio, estimated from minimal–maximal stimulation of EPSCs in brain slices (Case et al., 2011; Felix and Magnusson, 2016; Lee et al., 2016).

The SPON exhibited evidence for heterogeneity of excitatory inputs in 26% of the neurons in our sample. In addition to the octopus cell input documented here, a second excitatory input may arise from planar multipolar cells, which project out of the CN with thin axons via the VAS (Thompson and Thompson, 1991; Schofield, 1995, Doucet and Ryugo, 2003, 2006; although see remarks above for possibilities of spread of the tracer). However, we did not observe consistent labeling of planar multipolar cells or thin fibers in the VAS, in contrast with the previous findings. Another possible source of excitation may be the reciprocal connections between the SPON and TLCv (Saldaña et al., 2007; Viñuela et al., 2011). The TLCv provides descending input to SPON and many of its neurons express the vesicular glutamate transporter 2 (Aparicio and Saldaña, 2014). Whether the TLCv glutamatergic projection only targets a subclass of SPON neurons (Felix et al., 2013) remains to be investigated. For the majority (74%) of neurons, however, our findings are compatible with a single excitatory input to SPON neurons. The extremely low convergence of excitatory fibers with high release probability, which evoke an all-or-nothing EPSC in SPON neurons upon electrical stimulation in young adults (Felix and Magnusson, 2016), supports the conclusion that the majority of the SPON neurons receive a single strong excitatory input from octopus cells (Oertel et al., 1990) throughout the development of hearing.

### The SPON conveys temporal sound features to the midbrain

The fact that octopus cells provide a single strong excitatory input to both the SPON (this study; Felix and Magnusson, 2016) and the VNLL (Adams, 1997; Berger et al., 2014; Caspari et al., 2015) binds these brainstem areas together functionally. Indeed, there are several striking similarities documented between these two octopus cell-receiving nuclei (Pollak et al., 2011). In terms of their postsynaptic cellular properties recorded *in vitro*, neurons in both SPON (Felix et al., 2013) and VNLL (Caspari et al., 2015) respond with a single “onset” spike upon depolarization. Moreover, both neuronal populations undergo very similar developmental adjustments of their membrane time constants, resulting in equally fast kinetics and enhanced spiking precision (SPON: Felix et al., 2013; VNLL: Franzen et al., 2015). Their output is purely inhibitory and both types of neurons target the IC (SPON: Saldaña and Berrebi, 2000; Saldaña et al., 2009; VNLL: Saint Marie et al., 1997; Zhang et al., 1998; Riquelme et al., 2001; Caspari et al., 2015). Physiological *in vivo* recordings in both the SPON (Kulesza et al., 2003; Kadner and Berrebi, 2008; Felix et al., 2011, 2012) and VNLL (Batra and Fitzpatrick, 2002; Nayagam et al., 2005; Zhang and Kelly, 2006; Recio-Spinoso and Joris, 2014) have emphasized the strong response to temporal features of the sound stimuli. A temporally sharp but broadly tuned onset response (SPON: Felix et al., 2013; VNLL: Ritz and Brownell, 1982; Nayagam et al., 2006; Recio-Spinoso and Joris, 2014) results in well-timed inhibitory inputs to target neurons, which potentially could sharpen the acoustic feature binding in the IC.

Recently, we obtained topographically paired recordings from the SPON and the IC and found that selective blockade of SPON-derived inhibition led to enhanced segmentation of complex sounds in the IC (Felix et al., 2015). Inhibition from SPON may also contribute to facilitation of IC responses to harmonically related frequencies (Akimov et al., 2017), which is a mechanism that presumably enhances the perception of natural mouse calls (Ehret and Riecke, 2002). Strong inhibition from the SPON and VNLL that marks the onset of a broadband natural sound via octopus cell activation could deactivate local interneurons, providing inhibitory side fields to IC neurons and, thereby, achieve spectral facilitation within the same neural critical band (Ehret and Merzenich, 1988; Schreiner and Langner, 1997; Akimov et al., 2017). Simultaneously, slightly temporally dispersed inhibition from SPON and VNLL may sharpen the time window of integration of the spectral peaks (Nayagam et al., 2006; Mayko et al., 2012). This dual mechanism arising in brainstem areas processing preferentially monaural temporal information would be suitable to enhance clear boundaries between acoustic objects (Gaub and Ehret, 2005), such as vowels (Shamma and Micheyl, 2010).

The question remains whether the octopus cell projection into the SPON *en route* to the VNLL plays a significant role in human hearing. Comparative studies of the cat and primate (including humans) superior olivary complex reveal that the periolivary region, which contains the SPON, grows and expands in the medio-rostral plane compared to smaller mammals (Strominger et al., 1977; Moore, 1987, 2000; Bazwinsky et al., 2003, 2005; Ito et al., 2015). At the same time, the PVCN and MNTB, which provide the main inputs to the SPON and VNLL, are less prominent in humans (Moore and Osen, 1979; Moore, 1987). These facts have been difficult to reconcile with the expansion of presumptive auditory pathways that convey temporal information (Kulesza and Grothe, 2015). However, there is a striking similarity of the auditory cell types between species, irrespective of their exact location (Moore and Osen, 1979; Moore, 1987; Bazwinsky et al., 2003; Kulesza, 2014). Presumably these cells retain their specific inputs, although the cytoarchitectural borders are less well-defined in humans (Moore and Osen, 1979; Moore, 1987; Kulesza and Grothe, 2015). Based on these facts, we propose that octopus cells convey broad-band temporal information via IAS fibers that diverge and excite SPON and VNLL to form a temporally robust acoustic pathway in the brain enabling the detection of temporal features of speech.

## Author contributors

AM conceived the study. AM, BG, and ES contributed to the experimental design. RF, AM, MG, and SL acquired and BG, RF, MG, and ES analyzed the data. AM, BG, RF, MG, and ES contributed to the interpretation of the results. AM, RF, BG, and MG wrote the manuscript. All authors had full access to the data and take responsibility for the integrity and accuracy of the results.

### Conflict of interest statement

The authors declare that the research was conducted in the absence of any commercial or financial relationships that could be construed as a potential conflict of interest. The reviewer KM and handling Editor declared their shared affiliation, and the handling Editor states that the process nevertheless met the standards of a fair and objective review.

## Supplementary material

Supplementary Figure 1 **The EPSC parameter 10–90% Rise Time does not give rise to EPSC shape clustering here. Same than Figures 4B,D with clustering using the scatter plots of mEPSC decay time vs. 10–90% rise time. Distribution peaks disappear and only one cluster is found by the algorithm in all cases**.
