# Supplementary figures and images for: Octopus Cells in the Posteroventral Cochlear Nucleus Provide the Main Excitatory Input to the Superior Paraolivary Nucleus
Source: Front Neural Circuits. 2017 May 31;11:37. doi: 10.3389/fncir.2017.00037 (PMC5449481; doi:10.3389/fncir.2017.00037)

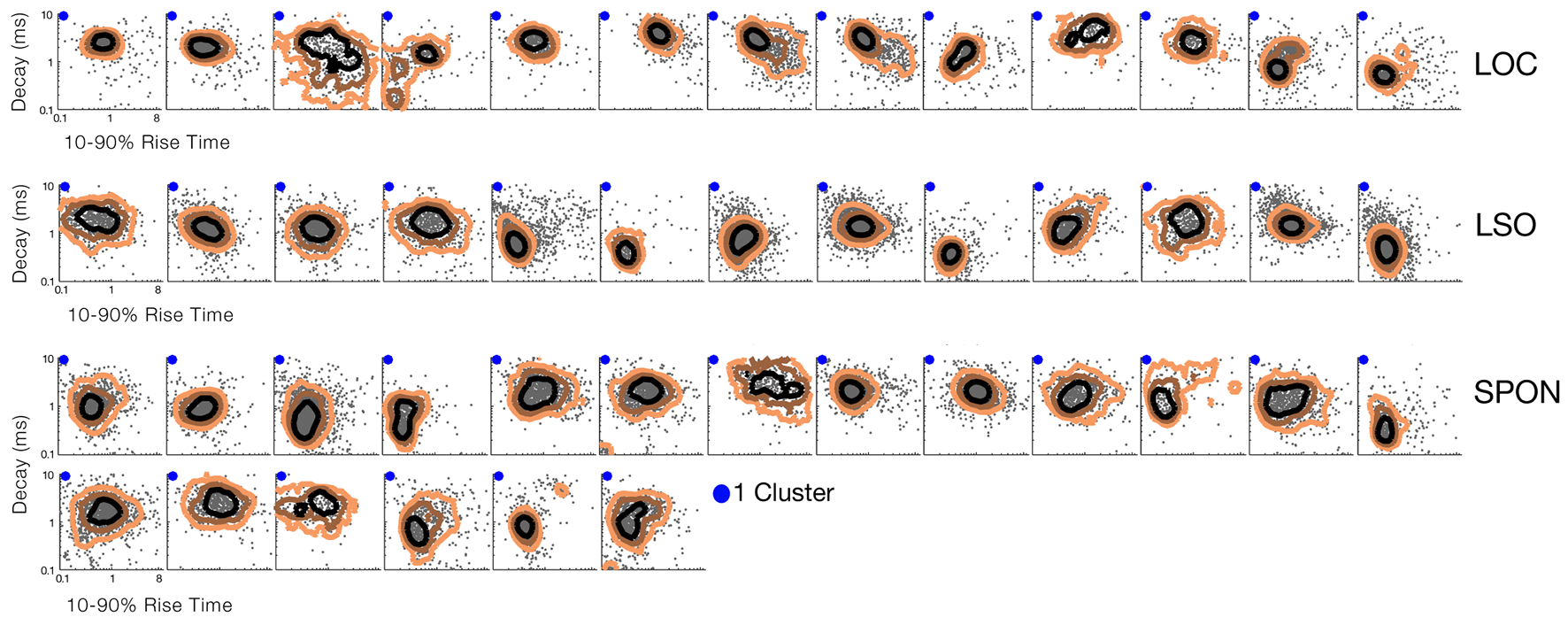

Supplement: Supplementary Figure 1 — The EPSC parameter 10–90% Rise Time does not give rise to EPSC shape clustering here. Same than Figures 4B,D with clustering using the scatter plots of mEPSC decay time vs. 10–90% rise time. Distribution peaks disappear and only one cluster is found by the algorithm in all cases. [file Image1.tif]
